# The Possible Role of Rosuvastatin Therapy in HFpEF Patients—A Preliminary Report

**DOI:** 10.3390/diagnostics14222579

**Published:** 2024-11-16

**Authors:** Tomasz Urbanowicz, Ievgen Spasenenko, Marta Banaszkiewicz, Anna Olasińska-Wiśniewska, Aleksandra Krasińska-Płachta, Andrzej Tykarski, Krzysztof J. Filipiak, Zbigniew Krasiński, Beata Krasińska

**Affiliations:** 1 Cardiac Surgery and Transplantology Department, Poznan University of Medical Sciences, 61-701 Poznan, Poland; 2 Department of Hypertensiology, Angiology and Internal Medicine, Poznan University of Medical Sciences, 61-701 Poznan, Poland; 3 Chair and Department of Pulmonary Circulation, Thromboembolic Diseases and Cardiology, Centre of Postgraduate Medical Education, European Health Centre, 05-400 Otwock, Poland; 4 Department of Vascular, Endovascular Surgery, Angiology and Phlebology, Poznan University of Medical Science, 61-701 Poznan, Poland; 5 Department of Ophthalmology, Poznan University of Medical Sciences, 61-701 Poznan, Poland; 6 Institute of Clinical Science, Maria Sklodowska-Curie Medical Academy, 00-136 Warsaw, Poland

**Keywords:** HFpEF, statin, BMI, rosuvastatin, sex

## Abstract

Background: An increasing number of heart failure with preserved ejection fraction (HFpEF) syndromes has been reported in tandem with increasing age and burdens of obesity and cardiometabolic disorders. Identifying possible risk and modulatory HFpEF factors has significant epidemiological and clinical value. This study aimed to assess the prevalence of echocardiographic diagnostic criteria of left ventricular dysfunction in patients with chronic coronary syndrome depending on rosuvastatin therapy. Method: There were 81 (33 (41%) male) consecutive patients with a median age of 70 (62–75) years, presenting with stable heart failure symptoms according to the New York Heart Association (NYHA) classification I to III. They presented with chronic coronary syndrome and were hospitalized between March and August 2024. Patients were divided according to the type of long-term lipid-lowering therapy into patients with rosuvastatin and with other statin therapy. The echocardiographic analysis based on diastolic dysfunction evaluation was performed on admission and compared with demographical, clinical, and laboratory results. Results: In the multivariable model for diastolic dysfunction prediction in the analyzed group based on three echocardiographic parameters, septal E’ below 7 cm/s, lateral E’ below 10 cm/s, and LAVI above 34 mL/m^2^, the following factors were found to be significant: sex (male) (OR: 0.19, 95% CI: 0.04–0.83, *p* = 0.027), obesity (defined as BMI > 30) (OR: 12.78, 95% CI: 2.19–74.50, *p* = 0.005), and rosuvastatin therapy (OR: 0.09, 95% CI: 0.02–0.51, *p* = 0.007). Conclusions: Rosuvastatin therapy can be regarded as a possible protective therapy against left ventricular diastolic dysfunction in chronic coronary syndrome.

## 1. Introduction

An increasing number of heart failure with preserved ejection fraction (HFpEF) syndromes has been reported in tandem with increasing age and burdens of obesity and cardiometabolic disorders [1]. It represents a heterogeneous group of pathologies classified as one condition requiring diagnostic scrutiny and pathophysiological-based phenotyping to individualize the therapy. The abnormality in left ventricular (LV) diastolic function is reported to display multifaceted limitations in cardiovascular function [2]. An impairment in left ventricular relaxation and viscoelastic chamber stiffness lead to clinical symptoms. A microvascular dysfunction may exaggerate subendocardial ischemia, impairing ventricular shortening during stress, while an increased myocardial oxygen supply is required, provoking clinical symptoms [3].

Chronic coronary syndrome describes the patients’ clinical manifestation that is accompanied by significant atherosclerosis in half of the patients [4]. A higher prevalence of myocardial ischemia with no obstructive coronary arteries is reported in women [5]. The pathophysiological background relates to epicardial coronary vascular and microvascular dysfunction. Chronic and acute coronary syndromes with non-obstructive epicardial disease are increasingly recognized in the clinical arena and are claimed to be associated with HFpEF [6]. So far, the appropriate primary and secondary prevention strategies have not yet been clearly established.

Statins are β-hydroxy β-methylglutaryl coenzyme A reductase (HMGCR) inhibitors that effectively modify dyslipidemic conditions and exhibit pleiotropic effects [7]. The pleiotropic effects are manifested by immunomodulation, anti-inflammatory properties, and antioxidant and anti-thrombotic action [8]. Rosuvastatin is a fully synthetic statin with a well-confirmed clinical effect on the cardiovascular system [9,10]. Previous reports have demonstrated the beneficial effects of rosuvastatin in reversing aortic remodeling in animal studies [11], through the increased expression of endothelial nitric oxide synthase and plasma nitrite/nitrate levels, apoptosis process suppression, and the upregulation of gap-junction complex connexin-43 both in media and the endothelium [12].

In large trials, rosuvastatin sufficiently achieved therapeutic goals, including low-density lipoprotein (LDL) cholesterol and triglyceride (TG) reduction, with an increase in high-density lipoprotein (HDL) cholesterol [13,14]. Moreover, rosuvastatin use was associated with lower LDL cholesterol levels compared with atorvastatin in patients with coronary artery disease [15] and diabetes mellitus (DM) [16]. In the Statin Therapies for Elevated Lipid Levels compared Across doses to the Rosuvastatin (STELLAR) trial [17], the efficacy of rosuvastatin, atorvastatin, simvastatin, and pravastatin was compared over 6 weeks of treatment, favoring rosuvastatin for LDL and TG lowering. Similarly, the beneficial effect of rosuvastatin was proved in a 12-week treatment [18]. Notably, statins differ in their optimal doses for their efficacy in lipid reduction. The VOYAGER meta-analysis [19] highlighted that reductions in LDL and non-HDL levels are achieved at lower doses of rosuvastatin than atorvastatin and simvastatin. However, a recent meta-analysis [20] of eight randomized clinical trials with a total of 8771 patients treated with rosuvastatin vs. atorvastatin did not reveal differences in clinical outcomes, such as all-cause death, cardiovascular death, myocardial infarction, stroke, and revascularization, or major adverse cardiovascular events (MACEs). This result points out the significance of studying the efficacy of lipid-lowering potential together with clinical outcomes.

The aim of this study was to assess the prevalence of echocardiographic diagnostic criteria of left ventricular dysfunction in patients with chronic coronary syndrome depending on rosuvastatin therapy.

## 2. Materials and Methods

### 2.1. Patients

There were 81 (33 (41%) male) consecutive patients with a median age of 70 (62–75) years, presenting with stable heart failure symptoms according to the New York Heart Association (NYHA) classification I to III. They presented with chronic coronary syndrome and were admitted to the internal medicine and hypertensiology department between March and August 2024. They were referred for hospitalization due to anginal symptoms according to the Canadian Cardiovascular Society (CCS) class I to III. Seventy-three (90%) patients were diagnosed with the co-existence of arterial hypertension. Seventy-seven (95%) patients presented with anginal symptoms on exertion and four (5%) more with an anginal equivalent. Seventy-six (94%) patients had a history of dyslipidemia, and nine patients (11%) had diabetes. None of the patients presented with atrial fibrillation.

Exclusion criteria included a left ventricular ejection fraction (LVEF) below 50% and valvular diseases.

A heart failure diagnostic algorithm [21] was used to describe the study group. All patients presented (1) symptoms and signs of heart failure (dyspnea, fatigue) and (2) an LVEF ≥ 50%. The consecutive investigation included an echocardiographic assessment of the evidence of structural and/or functional abnormalities revealing left ventricular diastolic dysfunction and laboratory analysis of natriuretic peptides. Subsequently, patients were further assessed and classified depending on the fulfillment of echocardiographic criteria thresholds.

Table 1 presents the characterization of the whole group. Patients were further divided according to the type of lipid-lowering therapy into patients with rosuvastatin and with other statin therapy. Ezetimibe was equally common in both subgroups. Only patients with permanent statin therapy (at least 6 months) were included in the analysis.

### 2.2. Methods

#### 2.2.1. Laboratory Tests

All venous blood samples were collected on admission using a routine hematology analyzer (Sysmex Europe GmbH, Norderstedt, Germany).

#### 2.2.2. Echocardiography

Transthoracic echocardiography was performed in each patient by the same echocardiographer using Vivid e95 (Vingmed Ultrasound, GE Company, Cincinnati, OH, USA) according to the same study protocol based on the current guidelines for the diagnosis and management of heart failure [22,23]. We assessed the left ventricular contractility and LVEF, septal and lateral e’, and relative wall thickness (RWT). The left atrial maximal volume was obtained from apical 4-chamber and 2-chamber views at end-systole and then normalized to body surface area (BSA) to calculate the left atrial volume index (LAVI). Diastolic dysfunction was diagnosed based on combined predictors reaching at least 5 points according to current guidelines [14]. Fulfilling all three parametric criteria (two functional and one structural) was considered sufficient for diastolic dysfunction diagnosis. The following combined parameters were used in the analysis: septal e’ below 7 cm/s + lateral e’ below 10 cm/s and RWT > 0.42 or septal e’ below 7 cm/s + lateral e’ below 10 cm/s + LAVI above 34 mL/m^2^.

#### 2.2.3. Coronary Angiography

The angiograms were performed according to a planned schedule in the reference hemodynamic center, and an experienced team evaluated the results. Any coronary artery disease was recognized as significant if an epicardial coronary artery stenosis of at least 50% of the lumen was observed.

### 2.3. Statistical Analysis

Since the data did not follow a normal distribution, the continuous variables are reported as medians and interquartile ranges (Q1–Q3). Categorical data are presented as numbers and percentages. The comparison of interval parameters between proximal and non-proximal groups was performed by the Mann–Whitney test. Categorical data were compared using a chi-square test of independence. A logistic regression analysis was performed to identify potential predictors of diastolic dysfunction. Both univariate and multivariable models were used. The multivariable model was assessed by the best subset method. The results are presented as the odds ratio (OR) and its 95% confidence intervals (95% CIs). Additionally, a receiver operating characteristic (ROC) curve was determined for the predicted factor of the significant model. Statistical analysis was performed using JASP statistical software (JASP Team; 2023. Version 0.18.1). *p* < 0.05 was considered statistically significant.

### 2.4. Bioethics Committee Approval

This study was performed according to the principles of Good Clinical Practice and the Declaration of Helsinki. It was approved by the Local Ethics Committee of the Medical University of Poznan (approval number: 875/22 on 3 November 2022). All patients gave their informed consent for inclusion in this study.

## 3. Results

Patients were divided into two subgroups based on long-term rosuvastatin therapy.

The rosuvastatin group (*n* = 54) and non-rosuvastatin group (*n* = 28) did not differ in terms of therapy duration, 18 (12–25) vs. 16 (7–41) months (*p* = 0.473), respectively.

In the rosuvastatin group, the mean (SD) daily dose was 21.5 (12.9) mg, including five (9%) patients treated with 5 mg per day, followed by sixteen (30%) patients treated with 10 mg/day and one (2%) with 15 mg daily. High-dose rosuvastatin therapy—20 mg, 30 mg, and 40 mg of rosuvastatin—was administered in fifteen (28%), two (4%), and fifteen (28%) patients, respectively.

The laboratory tests were conducted on admission, followed by transthoracic echocardiography and cine angiography. None of the laboratory tests, including whole blood count analysis and kidney and liver function tests, differentiated the groups. Moreover, the lipid profile was similar between subgroups, though the rosuvastatin group presented slightly lower LDL and higher HDL levels without statistical significance. The angiographies revealed no significant differences regarding normal angiograms (*p* = 0.153) or significant epicardial atherosclerosis requiring percutaneous interventions (*p* = 0.242), as presented in Table 2.

Echocardiographic examination revealed significant differences in the following parameters, as presented in Table 3: RWT > 0.42 (*p* = 0.010) and septal e’ (*p* = 0.018). The echocardiographic criteria for HFpEF were met in seven (9%) patients, including one (2%) in the rosuvastatin group and six (21%) in the non-rosuvastatin group (*p* = 0.006).

To present the possible factors of diastolic dysfunction in patients with HFpEF, multivariable models were created based on fulfilling diastolic dysfunction criteria [24].

**Figure 1:**
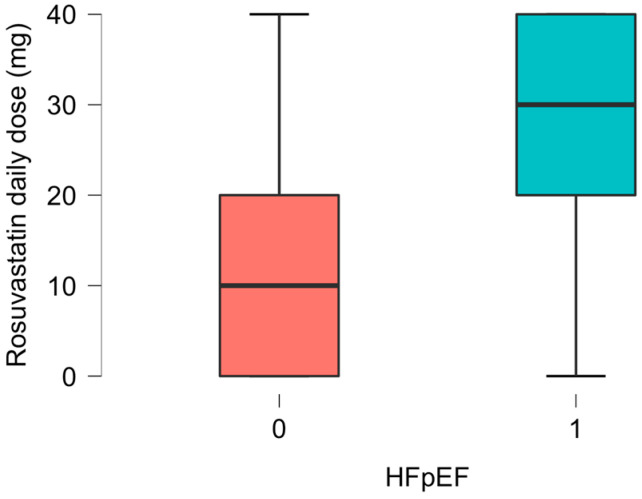
Group 0 which met the criteria for HFpEF diastolic dysfunction (septal e’ < 0.7 m/s + lateral e’ < 0.1 m/s + LAVI > 34 mL/m^2^) treated with a daily rosuvastatin dose of 10 (0–20) mg vs. Group 1 which did not fulfill the HFpEF criteria treated by a daily rosuvastatin dose of 30 (20–40) mg (*p* = 0.037).

We focused on a combination of three echocardiographic HFpEF diastolic dysfunction criteria, septal e’ below 7 cm/s, lateral e’ below 10 cm/s, and LAVI above 34 mL/m^2^, in relation to the daily dose of rosuvastatin (10 (0–20) mg vs. 30 (20–40) mg, *p* = 0.037), as presented in Figure 1.

**Figure 2:**
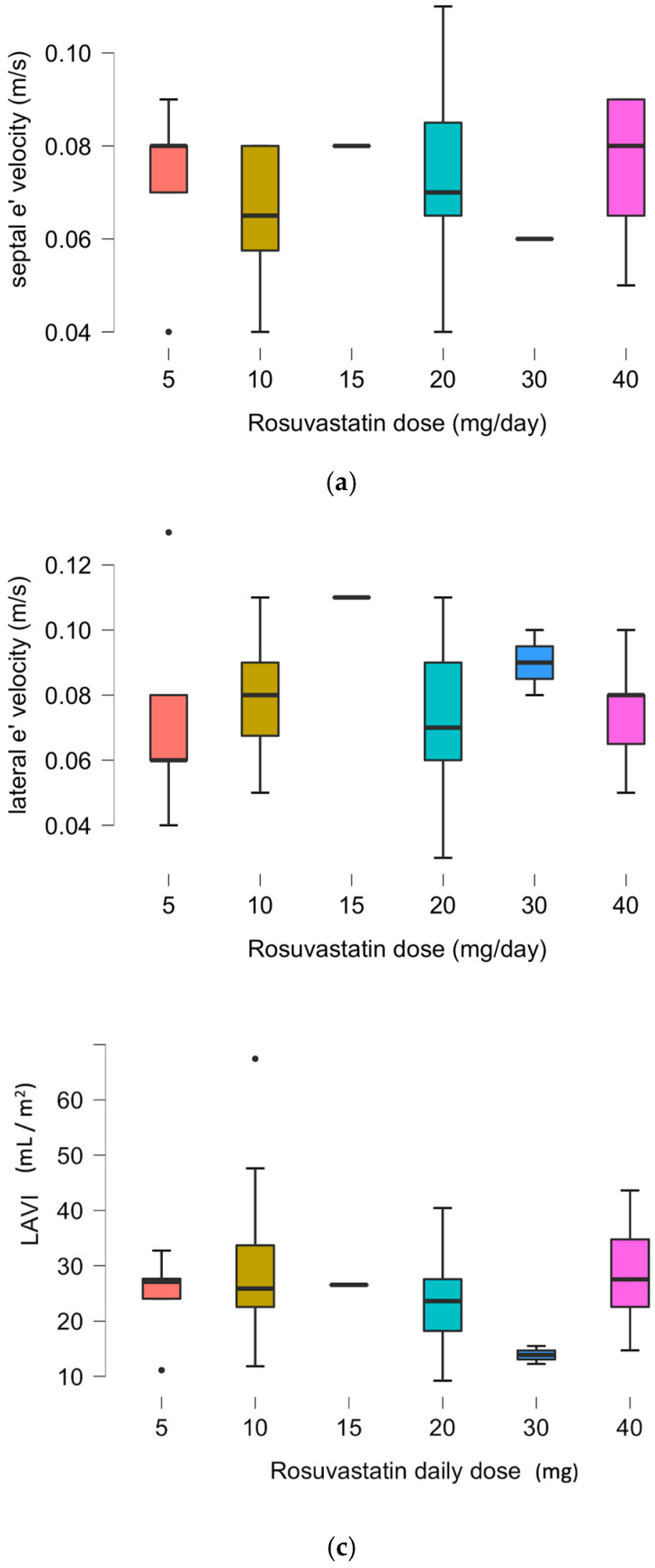
(**a**) Relation between daily rosuvastatin dose and septal e’ echocardiographic results. Rosuvastatin therapy: 5 (9%) pts—5 mg/day; 16 (30%) pts—10 mg/day; 1 (2%) pt—15 mg/day; 15 (28%) pts—20mg/day; 2 (4%) pts—30 mg/day; and 15 (28%) pts—40 mg/day. (**b**) Relation between daily rosuvastatin dose and lateral e’ echocardiographic results. Rosuvastatin therapy: 5 (9%) pts—5 mg/day; 16 (30%) pts—10 mg/day; 1 (2%) pt—15 mg/day; 15 (28%) pts—20 mg/day; 2 (4%) pts—30 mg/day; and 15 (28%) pts—40 mg/day. (**c**) Relation between daily rosuvastatin dose and LAVI echocardiographic results. Rosuvastatin therapy: 5 (9%) pts—5 mg/day; 16 (30%) pts—10 mg/day; 1 (2%) pt—15 mg/day; 15 (28%) pts—20 mg/day; 2 (4%) pts—30 mg/day; and 15 (28%) pts—40 mg/day.

There was no correlation between single echocardiographic parameters (septal e’ or lateral e’ or LAVI) and a daily dose of rosuvastatin in the rosuvastatin group, as presented in Figure 2.

Figure 2a–c show a comparison between daily rosuvastatin doses and separate echocardiographic parameters of HFpEF diastolic dysfunction (septal e’, lateral e’, LAVI).

### 3.1. Coronary Angiography

The coronary artery disease estimated by a lumen reduction of above 50% was found to be significant. The angiographic results revealed significant atherosclerosis located in the left descending artery in twelve (15%) patients (five (18%) in the non-rosuvastatin group vs. seven (13%) in the rosuvastatin group *p* = 0.533), followed by three (4%) patients with right coronary artery disease (two (7%) in the non-rosuvastatin group vs. one (2%) in the rosuvastatin group (*p* = 0.268)). Circumflex artery disease was noticed in two (4%) patients in the rosuvastatin group and one (4%) patient in the non-rosuvastatin group (*p* = 1.000).

The angiographic results were compared in diastolic dysfunction patients and compared with patients who did not meet the HFpEF criteria. Left descending artery (twelve (15%) patients) and right coronary artery disease (three (4%) patients) was noticed only in non-HFpEF patients. The atherosclerotic involvement of the circumflex artery was found in two (3%) patients with HFpEF and in one (1%) non-HFpEF patient.

### 3.2. Uni- and Multivariable Models for Diastolic Dysfunction Prediction

The uni- and multivariable models for diastolic dysfunction prediction were based on echocardiographic algorithms.

**Figure 3:**
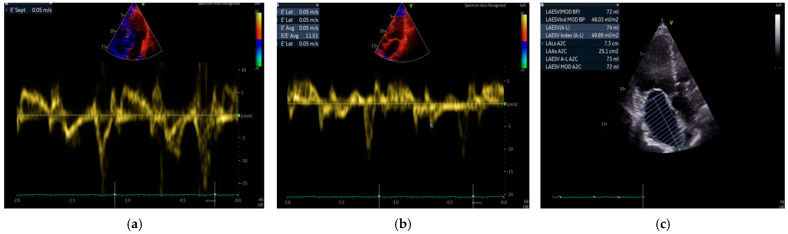
(**a**–**c**) Echocardiographic criteria for diastolic dysfunction diagnosis are based on three parameters: septal e’ (**a**), lateral e’ (**b**), and LAVI (**c**).

Uni- and multivariable models for left ventricular diastolic dysfunction diagnosed by three echocardiographic parameters in patients presenting with chronic coronary syndrome were created, as presented in Figure 3a–c.

In the univariable model, obesity (body mass index (BMI) above 30) (OR: 2.85, 95% CI: 1.01–8.07, *p* = 0.048) and rosuvastatin therapy (OR: 0.25, 95% CI: 0.09–0.71, *p* = 0.009) were found to be predictive of diastolic dysfunction.

The multivariable model indicated sex (male) (OR: 0.19, 95% CI: 0.04–0.83, *p* = 0.027), obesity (defined as BMI > 30) (OR: 12.78, 95% CI: 2.19–74.50, *p* = 0.005), and rosuvastatin therapy (OR: 0.09, 95% CI: 0.02–0.51, *p* = 0.007) as possible risk factors, as presented in Table 4.

**Figure 4:**
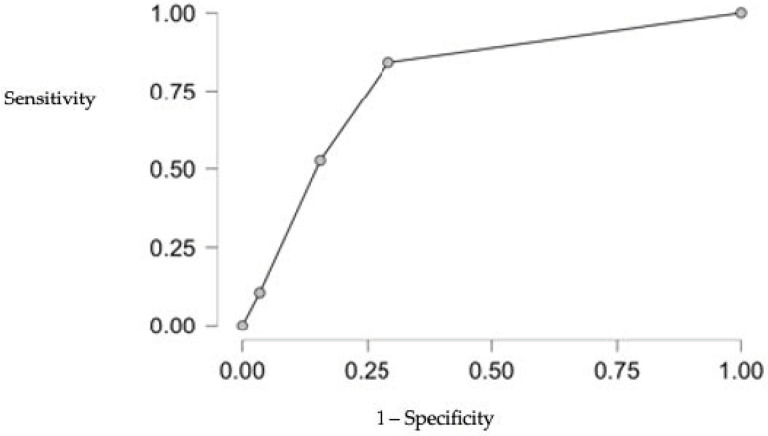
ROC curve analysis for HFpEF diastolic dysfunction prediction based on sex differences (higher predisposition in women), BMI, and inverse relation to long-term rosuvastatin therapy.

To confirm the adequacy of the obtained results, the ROC curve was created, characterized by an area under the curve (AUC) of 0.817 and an f-measure of 0.320 as presented in Figure 4.

## 4. Discussion

We present the results of our analysis indicating the possible beneficial role of long-term therapy with rosuvastatin on left ventricular diastolic function. The possible beneficial role of rosuvastatin was revealed in multivariable analysis, which was conducted with three models based on echocardiographic parameters describing diastolic dysfunction, such as single (septal e’), dual (septal and lateral e’), and a combination of three parameters (septal e’, lateral e,’ and LAVI).

HF with preserved ejection fraction accounts for at least half of the currently diagnosed HF patients. In this form, the left ventricle has abnormal diastolic function, and echocardiography is the most common and available non-invasive diagnostic tool [25,26].

Stress echocardiography combined with laboratory markers was found to be predictive of diastolic dysfunction [27]. Diastolic left ventricular (LV) filling depends on LV relaxation, compliance, and left atrial pressure. Elevated LV filling pressures are observed as a consequence of LV diastolic dysfunction. Diastolic dysfunction assessment by echocardiography is included in the criteria of diagnosis of heart failure with preserved ejection fraction. It enables differentiation from heart failure with a mildly reduced and reduced ejection fraction (HFmrEF and HFrEF, respectively) [14]. Despite the introduction of guidelines on diagnosing and managing heart failure, HFpEF remains underrecognized [1]. Since the diagnosis of HFpEF is challenging, no single echocardiographic parameter determines the disorder; rather, it is a combination of variables that should be recorded. Besides symptoms and preserved ejection fraction (≥50%), abnormalities consistent with the presence of diastolic dysfunction and raised left ventricular filling pressures are manifested. In particular, abnormalities in the left atrial (LA) size (LA volume index—LAVI), mitral E velocity, and septal e’ velocity showed their predictive value for the re-hospitalization rate and long-term survival [23,28,29]. Diastolic dysfunction, left atrial enlargement, and increased pulmonary pressure are hallmark patterns in HFpEF. In the PARAGON-HF (Prospective Comparison of ARNI With ARB Global Outcomes in HF With Preserved Ejection Fraction) trial, the prevalence of left ventricular hypertrophy was 21%, that of left atrial enlargement was 83%, that of an elevated E/e’ ratio was 53%, and that of pulmonary hypertension was 31% [21].

HFpEF is common in elderly women and men, ranging from 3% to 35% of the general population [30]. There are inconsistencies in the risk of HFpEF between male and female patients. Several studies underline that while the heart failure prevalence in overall is similar between sexes, women outnumber men in HFpEF. The lifetime risk of HFpEF is nearly double that of HFrEF in women, while the risk of both types is similar in men [31]. Men showed a higher risk of HFrEF, which could reflect the higher prevalence of coronary artery disease in the male population. Sex-based differences in mortality related to heart failure were presented in several analyses [32]. In a community-based cohort, an 11-year follow-up of PREVENT [27], male sex was associated with new-onset HFrEF, whereas females presented with new-onset HFpEF. In a cardiac surgical cohort of patients with coronary artery disease who underwent coronary revascularization, a significantly higher prevalence of diastolic dysfunction was revealed among females compared to males [33].

HFpEF is strictly related to co-morbidities, such as obesity, diabetes, coronary artery disease, atrial fibrillation, chronic pulmonary or kidney diseases, and arterial hypertension [23,34]. Thus, proper treatment of the disease influences the risk of HFpEF development and the course. Nevertheless, mortality and hospitalization rates remain high [35,36]. The possible association between worse outcomes, including overall mortality and repeated hospitalizations, is related to left ventricular diastolic dysfunction [37]. Moreover, despite the growing understanding of the syndrome’s pathophysiology, there has been limited success in developing specific treatments for patients with HFpEF. Novel HF pharmacology might positively impact this population’s long-term outcomes as the impact of sodium–glucose co-transporter 2 (SGLT-2) inhibitors on left ventricular strain parameters was postulated [38].

Our study confirms the aforementioned observations on the female sex and co-morbidity involvement in HFpEF. Female sex and BMI significantly influenced echocardiographic diastolic parameters’ combination occurrence.

The pathophysiology of HFpEF is multifaceted, including several disease-specific aspects of endothelial dysfunction and inflammatory activation. A growing body of literature supports increased interstitial fibrosis contributing to increased chamber stiffness [39]. Newer insights into myocardial remodeling have led to an interesting finding of apoptosis-resistant fibroblasts followed by an abnormal myocardial matrix [40].

Recently, impaired lipid handling, lipid accumulation in the myocardium, and subsequent lipotoxicity [41] have been postulated in the pathogenesis of HFpEF. Lipotoxicity accounts for lipid accumulation in non-adipose organs, resulting in oxidative stress, mitochondrial dysfunction, and apoptosis. Multiple studies have demonstrated intramyocardial lipid accumulation [42]. This may explain the phenomenon of the increased prevalence of HFpEF in women with coronary microvascular dysfunction and without obstructive changes in coronary arteries.

Abnormal lipid metabolism increases myocardial fat content and epicardial fat thickness and increases inflammation and oxidative stress, ultimately leading to cardiac diastolic dysfunction [43]. In a multicenter study, the atherogenic indices predicted diastolic dysfunction [44]. In animal models, a cholesterol-enriched diet induced left ventricular diastolic dysfunction, combined with evidence of cardiac inflammation and oxidative stress [45]. In Wu et al.’s [46] analysis, a relationship between adipocyte fatty acid-binding protein (AFABP) and left ventricular hypertrophy and diastolic development has been reported. The results of our study are especially important, as statin therapy discontinuation is common in clinical practice even following acute coronary syndromes [47,48].

Importantly, inflammation is profoundly included in the pathophysiology of coronary artery disease [49] and diabetes, and in non-ischemic cardiomyopathies’ background and relevant treatment strategies [50], lipid-lowering therapy may show beneficial effects in terms of primary and secondary prevention in HFpEF patients. Statins proved pleiotropic effects in reducing inflammation, suppressing immune–inflammatory reactions, reducing oxidative stress, and improving endothelium function [51].

In adults with coronary artery disease, rosuvastatin and atorvastatin presented comparable efficacy for the composite outcome of death, stroke, myocardial infarction, and any coronary revascularization. However, rosuvastatin was associated with lower LDL cholesterol levels [15]. In heart failure studies, the opinions on the benefits of statin use are contradictory. In patients with HFrEF, rosuvastatin failed to effectively reduce atherothrombotic- and heart failure-related events [52,53,54]. However, in repeated-event analysis, in the CORONA (Controlled Rosuvastatin Multinational Trial in Heart Failure) trial, rosuvastatin therapy reduced the risk of heart failure by approximately 15 to 20% [55]. High-dose rosuvastatin in chronic HF improved left ventricular performance [56]. Orkaby et al. [57] found reduced all-cause mortality, major adverse events, and hospitalization rates in patients with HFpEF without atherosclerotic cardiovascular disease.

The results of our analysis confirm the possible modulatory role of lipid-lowering therapies on left ventricular diastolic characteristics. Rosuvastatin therapy was shown to be a beneficial factor in LV diastolic function.

### Study Limitation

This is a single-center retrospective study performed on a limited number of patients. Further studies on a larger group of patients are required to confirm the hypothesis.

## 5. Conclusions

Rosuvastatin therapy can be regarded as a possible protective therapy against left ventricular diastolic dysfunction in chronic coronary syndrome. Further prospective studies are required to confirm the presented hypothesis.

## Figures and Tables

**Table 1:** Demographical and clinical characteristics.

Parameters	Study Group *n* = 81	Group 1 Non-Rosuvastatin Therapy *n* = 27	Group 2 Rosuvastatin Therapy *n* = 54	*p* 1 vs. 2
Demographic:				
Sex (M/F) (*n* (%))	33 (41)/48 (59)	9 (33)/19 (70)	24 (44)/30 (56)	0.343
Age (years) (median (Q1–Q3))	70 (62–75)	70 (64–75)	70 (62–75)	0.92
BMI (median (Q1–Q3))	27.8 (25.0–31.7)	27 (24.4–29.6)	28.5 (25.5–32.4)	0.085
BMI > 30 (*n* (%)) (mm) (*n* (%))	29 (36)	6 (22)	23 (43)	0.065
Functional status:				
CCS class (median (Q1–Q3))	1.5 (1.1)	1.5 (1.0)	1.5 (1.1)	0.095
NYHA class (median (Q1–Q3))	1.5 (0.2)	1.5 (0.3)	1.5 (0.2)	0.84
Co-morbidities:				
Arterial hypertension (*n* (%))	73 (90)	22 (82)	51 (94)	0.068
Dyslipidemia (*n* (%))	76 (94)	24 (89)	52 (96)	0.327
Diabetes mellitus (*n* (%))	9 (11)	2 (7)	7 (13)	0.462
COPD (*n* (%))	8 (10)	1 (4)	7 (13)	0.194
Active smoking (*n* (%))	22 (27)	5 (19)	17 (32)	0.292
Therapy prior to admission:				
B-blockers (*n* (%))	64 (79)	23 (85)	41 (76)	0.341
ARB (*n* (%))	20 (25)	6 (22)	14 (26)	0.722
ACE-I (*n* (%))	61 (75)	21 (78)	40 (74)	0.722
Loop diuretic (*n* (%))	17 (21)	7 (26)	10 (19)	0.447
MRA (*n* (%))	14 (17)	5 (19)	9 (17)	0.842
Rosuvastatin (*n* (%))	54 (67)	0 (0)	54 (100)	<0.001
Other statin (*n* (%))	27 (33)	27 (100)	0 (0)	<0.001
Ezetimibe (*n* (%))	27 (33)	7 (26)	21 (39)	0.253

Abbreviations: ACE-I—angiotensin-converting enzyme inhibitor; ARB—angiotensin receptor blocker; B-blockers—beta-blockers; BMI—body mass index; CCS—Canadian Cardiovascular Society; COPD—chronic obstructive pulmonary disease; F—female; M—male; MRA—mineralocorticoid receptor antagonist; Q—quartile; NYHA—New York Heart Association.

**Table 2:** Laboratory and coronary angiographic results.

Parameters	Group 1 Non-Rosuvastatin Therapy *n* = 27	Group 2 Rosuvastatin Therapy *n* = 54	*p*
Laboratory test results on admission:			
WBC (10^9^/L) (median (Q1–Q3))	7.01 (4.79–8.13)	6.74 (5.92–7.93)	0.378
Hb (mmol/L) (median (Q1–Q3))	8.7 (8.1–9.6)	8.9 (8.3–9.3)	0.952
Hct (%) (median (Q1–Q3))	43 (40–45)	42 (41–45)	0.782
Plt (10^3^/dL) (median (Q1–Q3))	221 (190–244)	244 (202–292)	0.039
ALAT (IU/L) (median (Q1–Q3))	27 (22–38)	25 (19–39)	0.499
Creatinine (umol/L) (median (Q1–Q3))	86 (80–108)	79 (71–108)	0.145
Glu (mmol/L) (median (Q1–Q3))	5.7 (5.5–6.2)	5.6 (5.2–6.0)	0.397
Hb1Ac (%) (median (Q1–Q3))	5.7 (5.5–6.0)	5.7 (5.4–5.9)	0.958
Lipoprotein (a) (mg/dL) (median (Q1–Q3))	9.0 (3.7–37.5)	10.1 (2.5–22.0)	0.951
Total cholesterol (mmol/L) (median (Q1–Q3))	4.41 (3.90–5.51)	4.04 (3.42–5.07)	0.109
HDL (mmol/L) (median (Q1–Q3))	1.35 (1.16–1.77)	1.55 (1.26–1.84)	0.764
LDL (mmol/L) (median (Q1–Q3))	2.40 (1.89–3.51)	1.90 (1.55–2.98)	0.077
LDL/HDL (median (Q1–Q3))	1.76 (1.02–2.34)	1.40 (0.99–1.90)	0.176
TG (mmol/L) (median (Q1–Q3))	1.01 (0.90–1.47)	1.24 (0.96–1.62)	0.188
UA (umol/L) (median (Q1–Q3))	398 (322–439)	352 (305–388)	0.058
BNP (pg/mL) (median (Q1–Q3))	178 (101–234)	163 (112–205)	0.127
Angiographic results:			
Normal angiograms (*n* (%))	14 (52)	38 (70)	0.14
Any angiographic disease (*n* (%))	13 (48)	16 (30)	0.14
Significant stenosis requiring PCI (*n* (%))	7 (26)	8 (15)	0.24

Abbreviations: ALAT—alanine aminotransferase; BNP—brain natriuretic peptide; Glu—glucose; Hb—hemoglobin; Hb1Ac—glycosylated hemoglobin; Hct—hematocrit; HDL—high-density lipoprotein cholesterol; LDL—low-density lipoprotein cholesterol; LDL/HDL—low-density lipoprotein/high-density lipoprotein cholesterol ratio; Q—quartile; PCI—percutaneous coronary intervention; Plt—platelet count; TG—triglyceride; UA—uric acid; WBC—white blood cell count.

**Table 3:** Echocardiographic results.

Parameters	Group 1 Non-Rosuvastatin Therapy *n* = 28	Group 2 Rosuvastatin Therapy *n* = 54	*p*
Dimensions:			
LVED (mm) (median (Q1–Q3))	44 (42–49)	48 (42–50)	0.488
LVES (mm) (median (Q1–Q3))	33 (31–38)	34 (30–38)	0.588
LVEDV (mm) (median (Q1–Q3))	48.9 (44.7–59.6)	54.7 (41.9–65.0)	0.627
LVESV (mm) (median (Q1–Q3))	30.0 (22.9–33.0)	26.1 (20.6–31.1)	0.304
LV performance:			
LVEF (%) (median (Q1–Q3))	54 (51–58)	56 (51–60)	0.189
Functional parameters:			
E velocity (mm) (median (Q1–Q3))	0.59 (0.55–0.78)	0.63 (0.55–0.77)	0.98
E/A (mm) (median (Q1–Q3))	0.91 (0.73–1.24)	0.85 (0.74–1.01)	0.375
Septal e’ (m/s) (median (Q1–Q3))	0.06 (0.05–0.07)	0.07 (0.06–0.08)	0.018 *
Lateral e’ (m/s) (median (Q1–Q3))	0.07 (0.06–0.09)	0.08 (0.06–0.09)	0.53
LV GLS (%) (median (Q1–Q3))	16 (13–19)	17.0 (15–19)	0.668
Structural parameters:			
LAVI (mL/m^2^) (median (Q1–Q3))	26.2 (22–35.4)	25.4 (19.3–32.4)	0.462
LVM index (g/m^2^) (median (Q1–Q3))	89.4 (79.2–117.1)	90.3 (72.1–115.8)	0.538
RWT (median (Q1–Q3))	0.52 (0.41–0.58)	0.42 (0.36–0.48)	0.010 *
Echocardiographic criteria for HFpEF (*n* (%)) (septal < 0.07 m/s + lateral < 0.10 m/s + LAVI > 34 mL/m^2^)	6 (21)	1 (2)	0.006 *

Abbreviations: E/A—ratio of peak velocity blood flow from left ventricular relaxation in early diastole (the E wave) to peak velocity flow in late diastole caused by atrial contraction (the A wave); GLS—global longitudinal strain; LAVI—left atrial volume index; LV—left ventricular; LVED—left ventricular end-diastolic diameter; LVEDV—left ventricular end-diastolic volume; LVEF—left ventricular ejection fraction; LVES—left ventricular end-systolic diameter; LVESV—left ventricular end-systolic volume; LVM—left ventricular mass; *n*—number; RWT—relative wall thickness; Q—quartile. * statistically significant.

**Table 4:** Uni- and multivariable models for diastolic dysfunction prediction based on three combined echocardiographic parameters (septal e’ below 7 cm/s + lateral e’ below 7 cm/s + LAVI above 34 mL/m^2^).

Parameters	Univariable			Multivariable		
	OR	95% CI	*p*	OR	95% CI	*p*
Demographical:						
Sex (male)	0.56	0.12–1.10	0.073	0.19	0.04–0.83	0.027
Age	1	0.94–1.06	0.955			
BMI > 30	2.85	1.01–8.07	0.048 *	12.78	2.19–74.50	0.005
NYHA	1.34	0.01–2.37	0.238			
Clinical:						
HA	1.06	0.29–5.84	0.95			
Dyslipidemia	0.5	0.08–3.22	0.466			
DM	0.8	0.15–4.18	0.788			
COPD	5.8	0.78–10.31	0.19			
Nicotine active	1.1	0.36–3.33	0.866			
Angiogram results:						
Any CAD	0.41	0.12–1.36	0.144			
Lipids:						
HDL						
LDL	1.01	0.35–3.53	0.86			
Lipoprotein	1.22	0.86–1.72	0.268			
Hb1Ac	1.01	0.99–1.02	0.413			
Echocardiography:						
LVM index	1.01	0.99–1.02	0.278			
LVEF	0.98	0.92–1.03	0.367			
Pharmacotherapy:						
B-blockers	1.83	0.47–7.12	0.386			
ARB/ACE-I	0.42	0.11–1.62	0.208			
Loop diuretic	1.78	0.56–5.63	0.325			
MRA	1.77	0.52–6.05	0.362			
Rosuvastatin	0.25	0.09–0.71	0.009 *	0.09	0.02–0.51	0.007
Ezetimibe	0.93	0.32–2.66	0.89			

Abbreviations: ACE-I—angiotensin-converting enzyme inhibitor; ARB—angiotensin receptor blocker; B-blockers—beta-blockers; BMI—body mass index; CAD—coronary artery disease; CI—confidence interval; COPD—chronic obstructive pulmonary disease; DM—diabetes mellitus; HA—arterial hypertension; Hb1Ac—glycosylated hemoglobin; HDL—high-density lipoprotein cholesterol; LDL—low-density lipoprotein cholesterol; LVEF—left ventricular ejection fraction; LVM—left ventricular mass; MRA—mineralocorticoid receptor antagonist; NYHA—New York Heart Association; OR—odds ratio. * statistically significant.

## Data Availability

The data will be shared for two years after publication on reasonable request via contacting the corresponding authors.
